# Weak Identification Robust Tests for Subvectors Using Implied Probabilities

**DOI:** 10.3390/e27040396

**Published:** 2025-04-08

**Authors:** Marine Carrasco, Saraswata Chaudhuri

**Affiliations:** 1 Department of Economics, University of Montreal, Montreal, QC H3T 1J4, Canada; 2 Department of Economics, McGill University, Montreal, QC H3A 0G4, Canada; saraswata.chaudhuri@mcgill.ca

**Keywords:** empirical likelihood, entropy, exponential tilting, projection based test, score test, weak instruments

## Abstract

This paper develops tests for hypotheses concerning subvectors of parameters in models defined by moment conditions. It is well known that conventional tests such as Wald, Likelihood-ratio and Score tests tend to over-reject when the identification is weak. To prevent uncontrolled size distortion and introduce refined finite-sample performance, we extend the projection-based test to a modified version of the score test using implied probabilities obtained by information theoretic criteria. Our test is performed in two steps, where the first step reduces the space of parameter candidates, while the second one involves the modified score test mentioned earlier. We derive the asymptotic properties of this procedure for the entire class of Generalized Empirical Likelihood implied probabilities. Simulations show that the test has very good finite-sample size and power. Finally, we apply our approach to the veteran earnings and find a negative impact of the veteran status.

## 1. Introduction

We are interested in developing tests for hypotheses concerning subvectors of an unknown parameter $\theta \in R^{d_{\theta }}.$ The true value of the parameter θ, denoted by $\theta^{0},$ satisfies a vector of moment conditions:

$$E\left[g\left(W_{i};\theta^{0}\right)\right]=0$$

where the vector $g\in R^{d_{g}}$ is known and $d_{g}\geq d_{\theta }.$ The moment conditions may stem from the first-order condition of the maximization of any criterion written as an expectation (for instance the expected utility in economics). They may also come from matching theoretical and empirical moments (see Section 4.1) or from instrumental variables (see Section 5). Other examples can be found in the book by Hall [1]. A particularity of our tests is that they are robust to weak identification.

To illustrate the notion of weak identification, consider the example of the linear instrumental variable regression:

$$y_{i}=X_{i}\theta^{0}+u_{i},$$

where the endogenous regressor $X_{i}$ is a scalar random variable related to the instruments $Z_{i}$ through the reduced-form equation

$$X_{i}=Z_{i}^{\prime }\Pi +V_{i}$$

where $E\left[Z_{i}u_{i}\right]=0$. Let $W_{i}={\left(y_{i},X_{i},Z_{i}^{\prime }\right)}^{\prime }$, and the moment condition corresponding to the orthogonality between $Z_{i}$ and $u_{i}$ is

$$g\left(W_{i};\theta \right)=Z_{i}\left(y_{i}-X_{i}\theta \right).$$

If Π is non-null and independent of *n*, the instruments $Z_{i}$ are strongly correlated with the endogenous regressor $X_{i}$, and hence the instruments are said to be strong. In that case, θ is identified in the sense that $Eg\left(W_{i};\theta \right)=0\Leftrightarrow \theta =\theta^{0}.$ Then, the GMM estimator is consistent with the $\sqrt{n}$ rate of convergence. When $\Pi =C/\sqrt{n}$, the correlations between $Z_{i}$ and $X_{i}$ go to zero, and the instruments are said to be weak (in the sense of Staiger and Stock [2]). In that case, the GMM estimator of θ is not consistent because $E\left[g(W_{i};\theta )\right]\to 0$ as n→∞ for all $\theta \neq \theta^{0}$ (with $\theta -\theta^{0}$ fixed). Then, the standard confidence intervals and tests are not reliable. In the nearly weak/semi-strong case, i.e., when $\Pi =C/n^{\phi }$ with 0<ϕ<1/2, the GMM estimator of θ is consistent with a slower rate of convergence than the usual $\sqrt{n}$ (see Antoine and Renault [3] and Andrews and Cheng [4]).

Based on a random sample $W_{i}$, i=1,2,…,n, the standard approach of inference is to conduct a Wald test based on the Generalized Method of Moments (GMM) estimator of θ or a score test. Wald tests have been shown to be inappropriate in the presence of weak identification (Dufour [5]). Moreover, the GMM-based score test proposed by Newey and West [6] is plagued by size distortions under common scenarios such as skewed moment vectors or models with weak identification; see the discussion in Wang and Zivot [7]. To improve the finite sample properties of this test, Chaudhuri and Renault [8] and Chaudhuri and Renault [9] propose to replace the uniform weights by implied probabilities obtained from an Information Theory criterion. These probabilities exploit the information from the model, namely that $E\left[g\left(W_{i},\theta \right)\right]=0$. So the implied probabilities ${\hat{\pi }}_{i}$ are selected such that the moments hold exactly:

$$\sum_{i=1}^{n}{\hat{\pi }}_{i}g\left(W_{i};\theta \right)=0.$$

However, given that the number of moments, $d_{g},$ is smaller than the sample size *n*, there is an infinity of possibilities for ${\hat{\pi }}_{i}$, i=1,2,…,n. The estimation of $\pi_{i}$ is an ill-posed problem. Which distribution should be used? A solution inspired from the entropy literature is to select the distribution obtained by minimizing the Cressie–Read divergence measure under the moment restrictions. Equivalently, one could also work with the Generalized Empirical Likelihood (GEL) that is characterized by the dual problem of this Cressie–Read divergence minimization. Two notable members of this class are the Empirical Likelihood estimator and the exponential tilting estimator (see Newey and Smith [10]). All these estimators can be viewed as Information Theory estimators (see Kitamura and Stutzer [11] and Golan [12]).

Chaudhuri and Renault [9] focus on tests for the entire parameter vector, i.e., $H_{0}:\theta =\theta_{0}$ and they show that implied probability-based score tests lead to improved finite sample properties compared to the conventional score test. In particular, they have better size control and remain powerful.

In this paper, we are concerned with testing the subsets of parameters $\theta_{1}\subset \theta$. More precisely, we want to test $H_{0}:\theta_{1}=\theta_{10}.$ The subset version of score tests suffers from important size distortion as shown by Guggenberger et al. [13]. To address this issue, we suggest to use the projection-based test developed by Chaudhuri and Zivot [14] coupled with the score test that includes the GEL implied probabilities.

The contribution of our paper is to provide a framework that opens up the possibility of applying any type of the Generalized Empirical Likelihood or Cressie–Read implied probabilities to the type of score tests discussed in Chaudhuri and Zivot [14]; see also Smith [15], Newey and Smith [10]. We derive the asymptotic properties of the resulting tests using the properties of the implied probabilities obtained in Chaudhuri and Renault [9] and generalized to include all the GEL estimators. Special care is taken to allow for weak identification. The simulations show that these tests perform well in terms of finite-sample size and exhibit strong power under the alternative. We complete the paper with an empirical illustration examining the effect of veteran status on earnings. Using our proposed test, we construct confidence intervals for the returns to veteran status on earnings, leveraging instrumental variables. This analysis, inspired by Chaudhuri and Rose [16], builds on the seminal natural experiment framework developed by Angrist [17] and Card [18], which earned them the 2021 Sveriges Riksbank Prize in Economic Sciences in Memory of Alfred Nobel. Our findings provide evidence of a negative impact of the veteran status on earnings.

The related literature is vast. The application of Information Theory measures to the estimation of econometric models goes back to Golan et al. [19], Kitamura and Stutzer [11], Imbens et al. [20], Smith [21], and Kitamura [22], among others. The use of the implied probabilities in the context of testing the hypothesis in the GMM setup was pioneered by Guggenberger and Smith [23] and further developed by Caner [24], Chaudhuri and Renault [8], and Chaudhuri and Renault [9]. Our current paper builds on this literature. Extensive research in econometrics has demonstrated that testing subsets of parameters in the face of commonly encountered problems such as weak identification is a much more difficult problem than testing the full parameter vector studied in Chaudhuri and Renault [9]; see Guggenberger et al. [13], Andrews et al. [25]. Chaudhuri and Zivot [14] provide an early contribution to the weak identification robust testing of subsets of parameters that is subsequently extended and refined in Andrews [26] and Andrews [27], and that is particularly suitable for application of the Information Theory. Time series extensions of generalized empirical likelihood are proposed by Otsu [28] and Guggenberger and Smith [29]. Recent papers have tried to develop most powerful tests for subvectors; see Guggenberger et al. [30], Kleibergen [31], and Kleibergen et al. [32]. In the current paper, we demonstrate that the application of Information Theory in the form of implied probabilities to the test of Chaudhuri and Zivot [14] for subsets of parameters delivers improved finite-sample performance.

The remainder of this paper is organized as follows. Section 2 describes the GMM framework and the implied probabilities in the context of the null hypothesis for subsets of parameters that is the focus of our interest. Section 3 discusses the score test for subsets of parameters and establishes its asymptotic properties. Section 4 provides evidence of the improved performance of this test using simulation results in empirically relevant settings. Section 5 includes the empirical application. Finally, Section 6 concludes. The main proofs are collected in Appendix A.

**Notation.** For a sequence $\left\{a_{n}\right\}$ of real numbers, $a_{n}=O\left(n^{k}\right)$ means $a_{n}/n^{k}\to c$ as n→∞ for some constant *c*; $a_{n}=o\left(n^{k}\right)$ means $a_{n}/n^{k}\to 0$ as n→∞. The notation $\overset{P}{\to }$ represents the convergence in probability as n→∞. If $\left\{a_{n}\right\}$ is a sequence of random variables, then $a_{n}=O_{p}\left(n^{k}\right)$ if $a_{n}/n^{k}-c_{n}\overset{P}{\to }0$ where $c_{n}$ is a deterministic sequence. Similarly $a_{n}=o_{p}\left(n^{k}\right)$ if $a_{n}/n^{k}\overset{P}{\to }0.$

## 2. Implied Probabilities for Hypothesis on Subsets of Parameters in the GMM Framework

### 2.1. Background

Let $W_{1},\ldots ,W_{n}$ be independent and identically distributed (i.i.d.) random variables. Let $g\left(W_{i};\theta \right):R^{d_{w}}\times \Theta \mapsto R^{d_{g}}$ be the $d_{g}$-dimensional moment vector, $W_{i}$ a $d_{w}$-dimensional random vector, $\Theta \subseteq R^{d_{\theta }}$ the parameter space, and let $d_{g}\geq d_{\theta }$. The dimensions $d_{w}$, $d_{g}$ and $d_{\theta }$ are fixed and hence do not depend on *n*. Suppose that we have a set of moment restrictions:

(1) $$E[g\left(W_{i};\theta^{0}\right)]=0$$

which holds for the true value of the parameter $\theta^{0}.$

Our goal is the testing of hypotheses on subsets of parameters, i.e., a subvector of θ. Without loss of generality, let $\theta ={\left(\theta_{1}^{\prime },\theta_{2}^{\prime }\right)}^{\prime }$, and let the null hypothesis of interest be

(2) $$H_{0}:\theta_{1}=\theta_{10}.$$

The parameter $\theta_{1}$ is the parameter of primary interest, while $\theta_{2}$ is the nuisance parameter.

The usual approach to tackle this problem consists in estimating θ by constrained GMM. Constrained estimators are obtained by imposing the null hypothesis. The estimator takes the form $\theta ={\left(\theta_{10}^{\prime },\theta_{2}^{\prime }\right)}^{\prime }$ that restricts $\theta_{1}$ by $H_{0}$ but lets the $\theta_{2}$ parameters be unrestricted. Given a first-step consistent estimator of θ, denoted $\bar{\theta }$, the constrained GMM estimator is a solution of

$${\hat{\theta }}_{GMM}=\arg\min_{\theta \in \Theta_{1}}Q_{n}\left(\theta \right)\equiv {\bar{g}}_{n}{\left(\theta \right)}^{\prime }{\hat{\Omega }}_{n}{\left(\bar{\theta }\right)}^{-1}{\bar{g}}_{n}\left(\theta \right)$$

where $\Theta_{1}$ is the set of elements ${\left(\theta_{1}^{\prime },\theta_{2}^{\prime }\right)}^{\prime }$ of Θ such that $\theta_{1}=\theta_{10}$, ${\bar{g}}_{n}\left(\theta \right):=\sum_{i=1}^{n}g\left(W_{i};\theta \right)/n$, and ${\hat{\Omega }}_{n}\left(\theta \right):=\frac{1}{n}\sum_{i=1}^{n}g\left(W_{i};\theta \right)g^{\prime }\left(W_{i};\theta \right).$ Let ${\bar{G}}_{n}\left(\theta \right)=\left(1/n\right)\sum_{i=1}^{n}\partial g\left(W_{i};\theta \right)/\partial \theta^{\prime }$. The score test proposed by Newey and West [6] is

$$LM_{n}={\left(\frac{\partial Q_{n}\left({\hat{\theta }}_{GMM}\right)}{\partial \theta }\right)}^{\prime }I_{n}^{-1}\left({\hat{\theta }}_{GMM}\right)\left(\frac{\partial Q_{n}\left({\hat{\theta }}_{GMM}\right)}{\partial \theta }\right)$$

where

(3) $$\begin{array}{ccc} \partial Q_{n}\left(\theta \right)/\partial \theta & = & {\bar{G}}_{n}{\left(\theta \right)}^{\prime }{\hat{\Omega }}_{n}{\left(\theta \right)}^{-1}{\bar{g}}_{n}\left(\theta \right)\;\mathrm{and}\\ I_{n}\left(\theta \right) & = & {\bar{G}}_{n}{\left(\theta \right)}^{\prime }{\hat{\Omega }}_{n}{\left(\theta \right)}^{-1}{\bar{G}}_{n}\left(\theta \right). \end{array}$$

According to Chaudhuri and Renault [8] and Chaudhuri and Renault [9], the poor finite sample properties of the score test can be improved by replacing the averages in ${\bar{G}}_{n}\left(\theta \right)$ and ${\hat{\Omega }}_{n}\left(\theta \right)$ by weighted sum using Information Theory. Instead of averaging using an equal weight 1/n, one should use the implied probabilities obtained from information theoretic criteria. The criterion considered is the Cressie–Read family.

The optimization problem solved by the implied probabilities ${\hat{\pi }}_{n}^{\left(\gamma \right)}\left(\theta \right)$ for θ∈Θ is

(4) $$\min_{\pi \in R^{n}}\frac{1}{\gamma (\gamma +1)}\sum_{i=1}^{n}\left[{\left(n\pi_{i}\right)}^{1+\gamma }-1\right]\;\;\mathrm{subject}\;\mathrm{to}\;\;\sum_{i=1}^{n}\pi_{i}=1\;\mathrm{and}\;\sum_{i=1}^{n}\pi_{i}g\left(W_{i};\theta \right)=0.$$

The objective function (4) is defined for any real γ, including the two limit cases γ→0 and γ→−1. γ=−1 corresponds to Empirical Likelihood (EL), and γ=1 corresponds to the Euclidean Empirical Likelihood (EEL), and γ→0 to the Kullback–Leibler Information Criterion (KLIC) that is consistent with Shannon’s entropy. The so-called Generalized Empirical Likelihood (GEL) estimator of θ is obtained by minimizing the criterion in (4) with respect to θ or alternatively by minimizing the dual problem based on the Lagrange multipliers associated with the constraints $\sum_{i=1}^{n}\pi_{i}g\left(W_{i};\theta \right)=0$; see Guggenberger and Smith [23] and Chaudhuri and Renault [9]. Here, however, the aim is not to estimate θ but to perform a test. Therefore, we need to go further than the aforementioned references and devise the two-step approach described in Section 3 to address the additional inferential issues of the uncontrolled over-rejection of the truth without an unnecessary loss in power.

### 2.2. Assumptions

Let us define

$$V\left(\theta \right):=Var\left(\sqrt{n}{\bar{g}}_{n}\left(\theta \right)\right)\;\mathrm{and}\;{\hat{V}}_{n}\left(\theta \right):=\frac{1}{n}\sum_{i=1}^{n}g\left(W_{i};\theta \right){\left[g\left(W_{i};\theta \right)-{\bar{g}}_{n}\left(\theta \right)\right]}^{\prime }.$$

We remark that this definition of ${\hat{V}}_{n}\left(\theta \right)$ corresponds to the appropriate estimator of $V\left(\theta \right)$ for the EEL estimator.

Consider a sequence of subsets $\Theta_{n}:n\geq 1$ of Θ containing $\theta^{0}$. $\Theta_{n}$ is a neighborhood of $\theta^{0}$ whose width depends on the identification strength in (1). Typically, $\Theta_{n}$ is narrower for strongly identified parameters and wider for weakly identified parameters.

We will maintain Assumptions 1 and 2 below to show the asymptotic equivalence of the implied probabilities for $\theta \in \Theta_{n}$; see Guggenberger and Smith [23] and Chaudhuri and Renault [9] for more discussion.

**Assumption** **1.**

*(i)*  *$\sup_{\theta \in \Theta_{n}}∥E\left[{\bar{g}}_{n}\left(\theta \right)\right]∥=O\left(\frac{1}{\sqrt{n}}\right)$.*
*(ii)*  *$\max_{1\leq i\leq n}\sup_{\theta \in \Theta_{n}}∥g(W_{i};\theta )∥=o_{p}\left(\sqrt{n}\right)$.*
*(iii)*  *$\sup_{\theta \in \Theta_{n}}∥g(W_{i};\theta )∥=O_{p}\left(1\right)$ for i=1,…,n.*
*(iv)*  *$\sup_{\theta \in \Theta_{n}}∥{\bar{g}}_{n}\left(\theta \right)-E\left[{\bar{g}}_{n}\left(\theta \right)\right]∥=O_{p}\left(\frac{1}{\sqrt{n}}\right)$.*

**Assumption** **2.**

*(i)*  *$\sup_{\theta \in \Theta_{n}}∥{\hat{\Omega }}_{n}\left(\theta \right)-V\left(\theta \right)∥=o_{p}\left(1\right)$, $\sup_{\theta \in \Theta_{n}}∥{\hat{V}}_{n}\left(\theta \right)-V\left(\theta \right)∥=o_{p}\left(1\right)$,*
*$\sup_{\theta \in \Theta_{n}}∥{\hat{\Omega }}_{n}^{-1}\left(\theta \right)-V^{-1}\left(\theta \right)∥=o_{p}\left(1\right)$ and $\sup_{\theta \in \Theta_{n}}∥{\hat{V}}_{n}^{-1}\left(\theta \right)-V^{-1}\left(\theta \right)∥=o_{p}\left(1\right)$.*
*(ii)*  *$0<\inf_{\theta \in \Theta_{n}}b_{\min}\left(\theta \right)<\sup_{\theta \in \Theta_{n}}b_{\max}\left(\theta \right)<+\infty$ where $b_{\min}\left(\theta \right)$ and $b_{\max}\left(\theta \right)$ stand for the smallest and largest eigenvalues, respectively, of V(θ).*

Assumption 1 is not restrictive if $\Theta_{n}$ is reduced to $\theta^{0}$ for all n. In that case, Assumptions 1(i) and 1(iii) are fulfilled by definition. Assumptions 1(ii) and 1(iv) follow from the fact that $g(W_{i};\theta^{0})$ is i.i.d. with zero mean and finite variance. Assumption 1(ii) is a consequence of the Borel–Cantelli Lemma. Assumption 1(iv) can be proved by the Lindeberg–Levy Central Limit Theorem. The validity of Assumption 1 when $\Theta_{n}$ is a local neighborhood of $\theta^{0}$, where the definition of local depends on the identification strength of θ, follows under mild conditions.

Regarding Assumption 2(i), it requires the uniform law of large numbers for the sample covariance matrix only. The two convergences on the first line of (i) are equivalent, provided Assumptions 1(i) and 1(iv) hold. The same is true for the convergence on the second line under the extra condition of Assumption 2(ii), which ensures that the population covariance matrix is positive definite and finite.

### 2.3. Properties of GEL Implied Probabilities

In this section, we investigate the properties of weighted sums based on implied probabilities. To do so, it is convenient to use the dual representation of the estimators introduced in Section 2.1.

Let $\rho \left(.\right)$ be a scalar function that is concave on its domain V, an open interval containing 0. The GEL class of estimators of $\theta^{0}$ is indexed by the function ρ and is defined as

$$\begin{array}{ccc} & & {\hat{\theta }}_{\rho ,n}:=\arg\min_{\theta \in \Theta }\underset{\lambda \in \Lambda_{n}\left(\theta \right)}{\sup}{\hat{Q}}_{\rho ,n}\left(\theta ,\lambda \right)\\ \mathrm{where} & & {\hat{Q}}_{\rho ,n}\left(\theta ,\lambda \right):=\frac{1}{n}\sum_{i=1}^{n}\rho \left(\lambda^{\prime }g\left(W_{i};\theta \right)\right)-\rho \left(0\right),\\ \;\mathrm{and} & & \Lambda_{n}\left(\theta \right):=\left\{\lambda \in R^{k}:\lambda^{\prime }g\left(W_{i};\theta \right)\in V,\forall \;i=1,\ldots ,n\right\}. \end{array}$$

Different choices of ρ(.) lead to different GEL estimators. The Continuous-Updating GMM or Euclidean Empirical Likelihood (EEL) estimator is a special case with ($\rho \left(v\right)=-{\left(1+v\right)}^{2}/2,V=R$) corresponding to γ→−1 in Equation (4), the Empirical Likelihood (EL) estimator (ρ(v)=ln(1−v),V=(−1,∞)) corresponds to γ=1, the exponential tilting (ET) estimator ($\rho \left(v\right)=-\exp\left(v\right),V=R$) to γ→0, etc., all of which satisfy Assumption ρ below.

**Assumption ρ:** (GEL function)
ρ:V↦R is a continuous function such that

(i) ρ is concave on its domain V, which is an open interval containing 0.
(ii) ρ is twice continuously differentiable on its domain. Defining $\rho_{r}\left(v\right):=\partial^{r}\rho \left(v\right)/\partial v^{r}$ for r=1,2 and $\rho_{r}:=\rho_{r}\left(0\right)$, let $\rho_{1}=\rho_{2}=-1$ (standardization for convenience).
(iii) There exists a positive constant *b* such that for each v∈V, $|\rho_{2}\left(v\right)-\rho_{2}\left(0\right)|\leq b\times |v|$ hold.

The desirable higher-order properties of the GEL estimators are due to the GEL first-order condition, which, assuming the differentiability of the moment vector g(w;θ) with respect to θ, is given by

$${\left[\sum_{i=1}^{n}\pi_{\rho ,i,n}\left({\hat{\theta }}_{\rho ,n}\right)G_{i}\left({\hat{\theta }}_{\rho ,n}\right)\right]}^{\prime }{\left[\sum_{i=1}^{n}\kappa_{\rho ,i,n}\left({\hat{\theta }}_{\rho ,n}\right)g\left(W_{i};{\hat{\theta }}_{\rho ,n}\right)g^{\prime }\left(W_{i};{\hat{\theta }}_{\rho ,n}\right)\right]}^{-1}{\bar{g}}_{n}\left({\hat{\theta }}_{\rho ,n}\right)=o_{P}\left(\frac{1}{\sqrt{n}}\right)$$

where for given θ and $\rho \left(.\right),\;{\bar{g}}_{n}\left(\theta \right):=\frac{1}{n}\sum_{i=1}^{n}g\left(W_{i};\theta \right),\;G_{i}\left(\theta \right):=\frac{\partial }{\partial \theta^{\prime }}g\left(W_{i};\theta \right),$

(5) $$\begin{array}{ccc} \lambda_{\rho ,n}\left(\theta \right) & : & =\arg\underset{\lambda \in \Lambda_{n}\left(\theta \right)}{\sup}{\hat{Q}}_{\rho ,n}\left(\theta ,\lambda \right), \end{array}$$

(6) $$\begin{array}{ccc} \pi_{\rho ,i,n}\left(\theta \right) & : & =\frac{\rho_{1}\left(\lambda_{\rho ,n}^{\prime }\left(\theta \right)g\left(W_{i};\theta \right)\right)}{\sum_{j=1}^{n}\rho_{1}\left(\lambda_{\rho ,n}^{\prime }\left(\theta \right)g\left(W_{j};\theta \right)\right)}\;:\;\mathrm{implied}\;\mathrm{probabilities}\;\mathrm{from}\;\mathrm{GEL},\\ \kappa_{\rho ,i,n}\left(\theta \right) & : & =\frac{\kappa_{\rho }\left(\lambda_{\rho ,n}^{\prime }\left(\theta \right)g\left(W_{i};\theta \right)\right)}{\sum_{j=1}^{n}\kappa_{\rho }\left(\lambda_{\rho ,n}^{\prime }\left(\theta \right)g\left(W_{j};\theta \right)\right)},\;\;\kappa_{\rho }\left(v\right):=\frac{\rho_{1}\left(v\right)+1}{v}\;\mathrm{if}\;v\neq 0,\kappa_{\rho }\left(0\right)=-1 \end{array}$$

Interestingly, the form of ρ(.) for EL leads to $\pi_{\rho ,i,n}\left(\theta \right)=\kappa_{\rho ,i,n}\left(\theta \right)$ for i=1,…,n. It is because of this along with the orthogonalization property of the implied probabilities $\pi_{\rho ,i,n}\left(\theta \right)$ (shown in Proposition 2 below) that the EL estimator has superior higher-order properties among the GEL class (see Newey and Smith [10]).

Note that Assumption ρ(iii) is a technical assumption needed only for the proofs. Now, we are able to establish some important results relative to the GEL implied probabilities.

**Proposition** **1.** *Let Assumptions 1, 2, and ρ hold. Then, for $\theta \in \Theta_{n}$:*

*(A)*  *$\lambda_{\rho ,n}\left(\theta \right)$ defined in (5) is such that $\lambda_{\rho ,n}\left(\theta \right)=-{\hat{\Omega }}_{n}^{-1}\left(\theta \right){\bar{g}}_{n}\left(\theta \right)+o_{P}\left(n^{-1/2}\right)$,*
*(B)*  *$\pi_{\rho ,i,n}\left(\theta \right)$ defined in (6) is such that for a given i=1,…,n,*
$$\pi_{\rho ,i,n}\left(\theta \right)=\pi_{EEL,i,n}\left(\theta \right)+o_{P}\left(n^{-3/2}\right),$$
  *where $\pi_{EEL,i,n}\left(\theta \right)$’s are the implied probabilities from EEL with the closed-form expression*
$$\pi_{EEL,i,n}\left(\theta \right)=\frac{1}{n}\left[1-{\left(g\left(W_{i};\theta \right)-{\bar{g}}_{n}\left(\theta \right)\right)}^{\prime }{\hat{\Omega }}_{n}^{-1}\left(\theta \right){\bar{g}}_{n}\left(\theta \right)\right]=\frac{1}{n}+O_{P}\left(n^{-3/2}\right).$$

**Remark** **1.** *It follows from (B) that the difference between the EEL and GEL implied probabilities is of a smaller order than that between the EEL implied probabilities and the naive empirical probabilities {1/n}. It may be tempting to argue that the use of the GEL implied probabilities to reweight the observations results in an equivalence up to one higher order. However, this result, in itself, is not sufficient for such a claim because (B) is not uniform in i=1,…,n. We provide a formal proof of this claim in Proposition 2.*

**Proposition** **2.** *Let Assumptions 1, 2, and ρ hold, and let θ be an arbitrary element of $\Theta_{n}$. Consider n i.i.d. realizations $\left\{Y_{1,n},\ldots ,Y_{n,n}\right\}$ of a d×1 random vector $Y_{n}$. Denote ${\bar{Y}}_{n}=\sum_{i=1}^{n}Y_{i,n}/n$. Assume that ${\bar{Y}}_{n}-E\left[{\bar{Y}}_{n}\right]\overset{P}{\to }0$, $\frac{1}{n}\sum_{i=1}^{n}\left(Y_{i,n}-{\bar{Y}}_{n}\right)\left[{\left(g\left(W_{i};\theta \right)-{\bar{g}}_{n}\left(\theta \right)\right)}^{\prime },Y_{i,n}^{\prime }\right]\overset{P}{\to }\left[\Omega_{Yg},\Omega_{YY}\right]$ (finite) and that*

$$\left(\begin{array}{c} \sqrt{n}\left({\bar{Y}}_{n}-E\left[{\bar{Y}}_{n}\right]\right)\\ \sqrt{n}\left({\bar{g}}_{n}\left(\theta \right)-E\left[{\bar{g}}_{n}\left(\theta \right)\right]\right) \end{array}\right)\overset{d}{\to }N\left(0,\left[\begin{array}{cc} \Omega_{YY} & \Omega_{Yg}\\ \Omega_{Yg}^{\prime } & V \end{array}\right]\right).$$

*Then, as n→∞, we have*

*(B)*  *$\left(\begin{array}{c} \sqrt{n}\sum_{i=1}^{n}\pi_{EEL,i,n}\left(\theta \right)\left(Y_{i}-E\left[{\bar{Y}}_{n}\right]\right)\\ \sqrt{n}\left({\bar{g}}_{n}\left(\theta \right)-E\left[{\bar{g}}_{n}\left(\theta \right)\right]\right) \end{array}\right)\overset{d}{\to }N\left(0,\left[\begin{array}{cc} \Omega_{YY}-\Omega_{Yg}V^{-1}\Omega_{Yg}^{\prime } & 0\\ 0 & V \end{array}\right]\right)$,*
*(B)*  *$\sqrt{n}\sum_{i=1}^{n}\pi_{\rho ,i,n}\left(\theta \right)\left(Y_{i}-E\left[{\bar{Y}}_{n}\right]\right)-\sqrt{n}\sum_{i=1}^{n}\pi_{EEL,i,n}\left(\theta \right)\left(Y_{i}-E\left[{\bar{Y}}_{n}\right]\right)\overset{P}{\to }0$.*

**Remark** **2.** *The proofs of Propositions 1 and 2 are given in the Appendix. Some of these results are already established in Chaudhuri and Renault [9]. However, the result for the ET estimator is not covered in Chaudhuri and Renault [9]. This is important because ET is the only GEL estimator fully consistent with Shannon’s entropy.*

Proposition 2 shows that the weighted average involving the implied probabilities is asymptotically independent of the average ${\bar{g}}_{n}\left(\theta \right)$. Replacing $Y_{i}$ by the first derivative of $g(W_{i};\theta )$ or by $g\left(W_{i};\theta \right)g{\left(W_{i};\theta \right)}^{T},$ one can deduce that the implied probability estimates of the Jacobian and variance are asymptotically independent of ${\bar{g}}_{n}\left(\theta \right)$. In the case of weak identification, this asymptotic independence of the estimated Jacobian (and estimated variance) with the moment vector leads to better finite-sample properties.

It follows from Proposition 2 that the use of the implied probabilities provides a more precise estimator of E[Y] since the asymptotic variance is smaller than Var(Y). The score test for the subsets of parameters that we will discuss now allows for weak identification that makes the use of implied probabilities necessary. Chaudhuri and Zivot [14] followed Kleibergen [33] and therefore implicitly used the EEL (Euclidean Empirical Likelihood) implied probabilities. Our paper opens up the possibility of using other implied probabilities for the same test for subsets of parameters, and demonstrates using simulations that other implied probabilities, such as those from EL, can provide significant improvement in its finite-sample performance.

## 3. Score Test for Subsets of Parameters Using the Implied Probabilities

### 3.1. Score Vector and Score Statistic Using the Implied Probabilities

Following Chaudhuri and Renault [9], we define the general score vector:

(7) $$l_{n}\left(\theta ,\pi^{G}\left(\theta \right),\pi^{V}\left(\theta \right)\right)=\left\{\sum_{i=1}^{n}\pi_{i,n}^{G}\left(\theta \right)G_{i}^{\prime }\left(\theta \right)\right\}{\left[\sum_{i=1}^{n}\pi_{i,n}^{V}\left(\theta \right)V_{i,n}\left(\theta \right)\right]}^{-1}\sqrt{n}{\bar{g}}_{n}\left(\theta \right)$$

where

$$G_{i}\left(\theta \right)=\frac{\partial g(W_{i};\theta )}{\partial \theta^{\prime }},\;\;V_{i,n}\left(\theta \right)=g\left(W_{i};\theta \right){\left(g\left(W_{i};\theta \right)-{\bar{g}}_{n}\left(\theta \right)\right)}^{\prime },$$

and $\pi_{i,n}^{G}\left(\theta \right)$ and $\pi_{i,n}^{V}\left(\theta \right)$ may be different but such that

(8) $$\pi_{i,n}^{G}\left(\theta \right),\pi_{i,n}^{V}\left(\theta \right)\in \left\{{\hat{\pi }}_{i,n}^{\left(\gamma \right)}\left(\theta \right);\gamma \in R\right\}\cup \left\{\frac{1}{n}\right\}.$$

The choice of $\pi_{i,n}^{G}\left(\theta \right)=\pi_{i,n}^{V}\left(\theta \right)=1/n$ leads to the standard GMM score statistic (3) as defined in Newey and West [6]. The choice of $\pi_{i,n}^{G}\left(\theta \right)={\hat{\pi }}_{i,n}^{\left(1\right)}\left(\theta \right)$ (EEL) and $\pi_{i,n}^{V}\left(\theta \right)=1/n$ leads to the K-statistic of Kleibergen [33]. The other choices in (8) cover the various score statistics of Guggenberger and Smith [23]. Importantly, note that $\pi_{i,n}^{G}\left(\theta \right)$ and $\pi_{i,n}^{V}\left(\theta \right)$ can be based on different γs, accommodating for hybrid GEL score statistics in the spirit of Schennach [34]. We refer the interested reader to Chaudhuri and Renault [9] for further discussion on the score vector.

Pretending that the parameters are all strongly identified, the natural estimator of the asymptotic variance of $l_{n}\left(\theta ,\pi^{G}\left(\theta \right),\pi^{V}\left(\theta \right)\right)$ would be

(9) $$I_{n}\left(\theta ,\pi^{G}\left(\theta \right),\pi^{V}\left(\theta \right)\right)=\left\{\sum_{i=1}^{n}\pi_{i,n}^{G}\left(\theta \right)G_{i}^{\prime }\left(\theta \right)\right\}{\left[\sum_{i=1}^{n}\pi_{i,n}^{V}\left(\theta \right)V_{i,n}\left(\theta \right)\right]}^{-1}\left\{\sum_{i=1}^{n}\pi_{i,n}^{G}\left(\theta \right)G_{i}\left(\theta \right)\right\}.$$

Using (9), the general score statistic based on the general score vector in (7) is given by

(10) $${\mathrm{LM}}_{n}\left(\theta ,\pi^{G}\left(\theta \right),\pi^{V}\left(\theta \right)\right)=l_{n}^{\prime }\left(\theta ,\pi^{G}\left(\theta \right),\pi^{V}\left(\theta \right)\right)I_{n}^{-1}\left(\theta ,\pi^{G}\left(\theta \right),\pi^{V}\left(\theta \right)\right)l_{n}\left(\theta ,\pi^{G}\left(\theta \right),\pi^{V}\left(\theta \right)\right).$$

It is now well known that if $\theta_{2}$ is weakly identified, then plugging in a GMM estimator of $\theta_{2}$ that is restricted by $H_{0}$ in (2) generally results in a badly over-sized test; see Andrews [26] for a comprehensive discussion. An alternative to such plug-in tests is the projection tests as in, for example, Dufour and Taamouti [35,36]. However, projection tests can be needlessly conservative.

Therefore, we adopt here the idea of the refined projection score test as in Chaudhuri [37], Zivot and Chaudhuri [38], Chaudhuri et al. [39], Chaudhuri and Zivot [14]. Our presentation can be adapted to the more sophisticated version of the aforementioned tests that was introduced in Andrews [26], but that is not performed here for simplicity and brevity.

To present the refined projection score test for the null hypothesis (2) on $\theta_{1}$, treating $\theta_{2}$ as the nuisance parameters, it will be useful to introduce the natural partition of $l_{n}\left(\theta ,\pi^{G}\left(\theta \right),\pi^{V}\left(\theta \right)\right)$ and $I_{n}\left(\theta ,\pi^{G}\left(\theta \right),\pi^{V}\left(\theta \right)\right)$ conformable to the partition of $\theta ={\left(\theta_{1}^{\prime },\theta_{2}^{\prime }\right)}^{\prime }$ as

(11) $$\begin{array}{cc} l_{n}\left(\theta ,\pi^{G}\left(\theta \right),\pi^{V}\left(\theta \right)\right)= & \left[\begin{array}{c} l_{1,n}\left(\theta ,\pi^{G}\left(\theta \right),\pi^{V}\left(\theta \right)\right)\\ l_{2,n}\left(\theta ,\pi^{G}\left(\theta \right),\pi^{V}\left(\theta \right)\right) \end{array}\right],\\ I_{n}\left(\theta ,\pi^{G}\left(\theta \right),\pi^{V}\left(\theta \right)\right)= & \left[\begin{array}{cc} I_{11,n}\left(\theta ,\pi^{G}\left(\theta \right),\pi^{V}\left(\theta \right)\right)\; & I_{12,n}\left(\theta ,\pi^{G}\left(\theta \right),\pi^{V}\left(\theta \right)\right)\\ I_{21,n}\left(\theta ,\pi^{G}\left(\theta \right),\pi^{V}\left(\theta \right)\right) & I_{22,n}\left(\theta ,\pi^{G}\left(\theta \right),\pi^{V}\left(\theta \right)\right) \end{array}\right],\\ l_{1.2,n}\left(\theta ,\pi^{G}\left(\theta \right),\pi^{V}\left(\theta \right)\right)= & \;l_{1,n}\left(.,.,.\right)-I_{12,n}\left(.,.,.\right)I_{22,n}^{-1}\left(.,.,.\right)l_{2,n}\left(.,.,.\right),\\ I_{11.2,n}\left(\theta ,\pi^{G}\left(\theta \right),\pi^{V}\left(\theta \right)\right)= & \;I_{11,n}\left(.,.,.\right)-I_{12,n}\left(.,.,.\right)I_{22,n}^{-1}\left(.,.,.\right)I_{21,n}\left(.,.,.\right) \end{array}$$

where the right-hand side of the last two lines above use $\left(.,.,.\right)$ to denote $\left(\theta ,\pi^{G}\left(\theta \right),\pi^{V}\left(\theta \right)\right)$ to avoid notational clutter. Using the notation in (11), it is straightforward to decompose the score statistic in (10) as follows:

(12) $${\mathrm{LM}}_{n}\left(\theta ,\pi^{G}\left(\theta \right),\pi^{V}\left(\theta \right)\right)={\mathrm{LM}}_{2,n}\left(\theta ,\pi^{G}\left(\theta \right),\pi^{V}\left(\theta \right)\right)+{\mathrm{LM}}_{11.2}\left(\theta ,\pi^{G}\left(\theta \right),\pi^{V}\left(\theta \right)\right)$$

where, borrowing the maximum-likelihood terminology from Cox and Hinkley [40],

$$\begin{array}{ccc} {\mathrm{LM}}_{2,n}\left(\theta ,\pi^{G}\left(\theta \right),\pi^{V}\left(\theta \right)\right) & = & l_{2,n}^{\prime }\left(\theta ,\pi^{G}\left(\theta \right),\pi^{V}\left(\theta \right)\right)I_{22,n}^{-1}\left(\theta ,\pi^{G}\left(\theta \right),\pi^{V}\left(\theta \right)\right)l_{2,n}\left(\theta ,\pi^{G}\left(\theta \right),\pi^{V}\left(\theta \right)\right),\\ {\mathrm{LM}}_{1.2,n}\left(\theta ,\pi^{G}\left(\theta \right),\pi^{V}\left(\theta \right)\right) & = & l_{1.2,n}^{\prime }\left(\theta ,\pi^{G}\left(\theta \right),\pi^{V}\left(\theta \right)\right)I_{11.2,n}^{-1}\left(\theta ,\pi^{G}\left(\theta \right),\pi^{V}\left(\theta \right)\right)l_{1.2,n}\left(\theta ,\pi^{G}\left(\theta \right),\pi^{V}\left(\theta \right)\right) \end{array}$$

are respectively the score statistic for $\theta_{2}$ and the efficient score statistic for $\theta_{1}$. The efficient score statistic ${\mathrm{LM}}_{1.2,n}\left(\theta ,\pi^{G}\left(\theta \right),\pi^{V}\left(\theta \right)\right)$ expressed at $\theta =\left(\theta_{10},\theta_{2}^{0}\right)$ can be seen as the C(α) statistic of Neyman [41] for testing $H_{0}:\theta_{1}=\theta_{10}.$ Interestingly, this test has, under standard regularity conditions, an asymptotic distribution that is invariant to the $\sqrt{n}$-local perturbation of $\theta_{2}$ from the truth $\theta_{2}^{0}$; see, for example, Bera and Bilias [42]. So the unknown nuisance parameter $\theta_{2}^{0}$ can be replaced by a $\sqrt{n}$-consistent estimator without altering the asymptotic distribution of the C(α) statistic.

Another important fact is that ${\mathrm{LM}}_{1.2,n}\left(\theta ,\pi^{G}\left(\theta \right),\pi^{V}\left(\theta \right)\right)$ can be constructed using any choice of implied probabilities (including 1/n) for the Jacobian or the variance matrix, which will now allow us to explore the improved performance of the refined projection score test idea for the null hypothesis $H_{0}:\theta_{1}=\theta_{10}$ in (2) with the use of these implied probabilities.

### 3.2. Refined Projection Score Test Using the Implied Probabilities

To test the null hypothesis $H_{0}:\theta_{1}=\theta_{10},$ we propose to use the refined projection score test as in Chaudhuri [37], Zivot and Chaudhuri [38], Chaudhuri et al. [39], Chaudhuri and Zivot [14] but with the accommodation for the various choice of implied probabilities. The test is conducted in two steps:

- **Step 1:** Construct a 100(1−τ)% confidence interval $C_{H_{0}}\left(\theta_{2},1-\tau \right)$ for $\theta_{2}$ under the restriction of the null hypothesis $H_{0}:\theta_{1}=\theta_{10}$. $C_{H_{0}}\left(\theta_{2},1-\tau \right)$ is a random subset of the parameter space $\Theta_{2}$ of $\theta_{2}$ and is defined as follows:
  $$C_{H_{0}}\left(\theta_{2},1-\tau \right)=\left\{\theta_{2}\in \Theta_{2}\;|\;n{\bar{g}}_{n}^{T}\left(\theta_{10},\theta_{2}\right){\left[{\hat{\Omega }}_{n}\left(\theta_{10},\theta_{2}\right)\right]}^{-1}{\bar{g}}_{n}\left(\theta_{10},\theta_{2}\right)\leq \chi_{d_{g}}^{2}\left(1-\tau \right)\right\}$$
  where $\chi_{a}^{2}\left(b\right)$ denotes the *b*-th quantile of a chi-square distribution with *a* degrees of freedom.
- **Step 2:** Reject the null hypothesis $H_{0}:\theta_{1}=\theta_{10}$ if either $C_{H_{0}}\left(\theta_{2},1-\tau \right)$ is empty or
  $$\underset{\theta_{2}\in C_{H_{0}}\left(\theta_{2},1-\tau \right)}{\inf}{\mathrm{LM}}_{1.2,n}\left(\theta_{10},\theta_{2},\pi^{G}\left(\theta_{10},\theta_{2}\right),\pi^{V}\left(\theta_{10},\theta_{2}\right)\right)\geq \chi_{d_{\theta_{1}}}^{2}\left(1-\alpha \right)$$
  where $d_{\theta_{1}}$ is the dimension of $\theta_{1}$. When deemed necessary, one should impose $\pi_{i,n}^{G}\left(\theta_{10},\theta_{2}\right)\neq 1/n$ following Kleibergen [33,43] to be robust to the weak identification of θ.

Step 1 corresponds to inverting the S-test of Stock and Wright [44]. In special cases, such as the linear instrumental variables regression with conditionally homoskedastic error, $C_{H_{0}}\left(\theta_{2},1-\tau \right)$ can be obtained analytically using closed-form formula presented in Dufour and Taamouti [35]. Moreover, Sun [45] provides a STATA command “twostepweakiv” with the “project” option to obtain confidence intervals for $\theta_{1}$ based on the version of this refined projection test from Chaudhuri and Zivot [14].

The difference between the refined projection test and the Newey and West [6], Kleibergen [33,43] or Guggenberger and Smith [23] score test is that the former performs a projection of ${\mathrm{LM}}_{1.2,n}\left(.\right)$ from $C_{H_{0}}\left(\theta_{2},1-\tau \right)$, while the latter plugs in an estimator of $\theta_{2}$ in ${\mathrm{LM}}_{n}\left(.\right)$ that makes ${\mathrm{LM}}_{2,n}\left(.\right)$ in (12) zero. This difference enables the refined projection test to guard against the uncontrolled over-rejection of a true $H_{0}$ under weak identification. All these tests are asymptotically equivalent under strong identification thanks to the C(α) form of ${\mathrm{LM}}_{1.2,n}\left(.\right)$.

On the other hand, the refinement provided by the refined projection test over the standard projection test principle is two-fold. First, the projection is performed from $C_{H_{0}}\left(\theta_{2},1-\tau \right)$ instead of from $\Theta_{2}$, as is performed by the latter. Second, the test statistic and critical values used are ${\mathrm{LM}}_{1.2,n}\left(.\right)$ and $\chi_{d_{\theta_{1}}}^{2}\left(\alpha \right)$ instead of ${\mathrm{LM}}_{n}\left(.\right)$ and $\chi_{d_{\theta }}^{2}\left(\alpha \right)$, as is performed by the standard projection score test. The restricted projection from $C_{H_{0}}\left(\theta_{2},1-\tau \right)$ instead of from $\Theta_{2}$ and the use of the smaller critical values based on the degrees of freedom $d_{\theta_{1}}$ instead of $d_{\theta }$ of the chi-squared distribution are what make the refined projection test more powerful than the standard projections tests.

Without the weak identification problem, the refined projection test is the efficient test in the sense of Newey and West [6]. The standard projection score test is less powerful. In presence of weak identification, both the standard projection score test and the refined projection score test guard against the uncontrolled over-rejection of the truth, while the Newey and West [6], Kleibergen [33,43] or Guggenberger and Smith [23] score tests do not do so.

The following proposition makes precise the statement about “uncontrolled over-rejection” and “efficient test” made above. For brevity, we list the technical assumptions Θ, SW, and D in the Appendix. These additional assumptions are essential for establishing the asymptotic properties of the refined projection test in Chaudhuri and Zivot [14] to which we refer the readers for the proof. Then, by appealing to the results in Propositions 1 and 2 that were obtained under Assumptions 1, 2, and ρ, the results stated in Proposition 3 follow directly.

**Proposition** **3.** *Let Assumptions 1, 2, ρ, and the three assumptions Θ, SW and D, stated in the Appendix, hold. Then, we obtain the following results for the refined projection score test using the implied probabilities in (8):*

*(i)*  *The asymptotic size of the test cannot exceed α+τ for any choice of α>0 and τ>0 with α+τ<1 under a restriction in (8) that $\pi_{i,n}^{G}\left(\theta \right)\in \left\{{\hat{\pi }}_{i,n}^{\left(\gamma \right)}\left(\theta \right);\gamma \in R\right\}$.*
*(ii)*  *If all elements of θ are strongly identified as in Newey and West [6], and $\theta_{10}=\theta_{1}^{0}+b/\sqrt{n}$, then the test with any given τ>0, such that $C_{H_{0}}\left(\theta_{2},1-\tau \right)$ is non-empty and is asymptotically equivalent to the infeasible efficient score test that rejects $H_{0}:\theta_{1}=\theta_{10}$ if ${\mathrm{LM}}_{1.2,n}\left(\theta_{10},\theta_{2}^{0},1/n,1/n\right)\geq \chi_{d_{\theta_{1}}}^{2}\left(\alpha \right)$.*

**Remark** **3.** *The tests discussed here involving various implied probabilities have the same first-order asymptotic properties as the test in Chaudhuri and Zivot [14]. Indeed, their asymptotic size cannot exceed α+τ, and if there is no problem of weak identification, then for any choice of τ (however small or large), these tests are asymptotically equivalent to the asymptotically efficient infeasible score test with asymptotic size α. So, with strong identification, the asymptotic size of this test is α, provided the first-step confidence interval is non-empty. The results in Chaudhuri and Renault [9] suggest that the use of the implied probabilities could lead to better properties in the finite samples. This is precisely what we find in the Monte Carlo experiment described below.*

## 4. Monte Carlo Experiment

The improvement in the finite-sample size properties of tests by the use of implied probabilities is well known. The characterization of the asymptotic size described in Proposition 3(i) of the refined projection test appeals to the Bonferroni inequality applied to the size properties of two full vector score tests. Guggenberger and Smith [23] and Chaudhuri and Renault [9] document evidence that the finite-sample size of the full vector score tests with various implied probabilities is similar to their nominal level under various scenarios involving different strengths of identification. This will be confirmed here in our simulations.

On the other hand, less attention has been paid to the matter of improvement in power; the work of Chaudhuri and Renault [9] is an exception but only when testing a full vector (θ and not $\theta_{1}$). However, there is a big difference between the power of a test for the full vector θ versus a test for subset of θ, and the main advantage of the refined projection test concerns its power. Therefore, we will primarily focus on the power properties of the refined projection score test for $\theta_{1}$, compared to that of the plug-in tests. Since the power properties of the plug-in tests are better understood when parameters θ are strongly identified (see Andrews [26]), we will maintain strong identification of θ in this section.

### 4.1. Design

In this section, we examine a model that is not subject to weak identification but is instead affected by large higher-order moments, leading to difficult estimation of the variance matrix. This is the same experiment as that considered in the unpublished manuscript by Chaudhuri and Renault [46]. We generate

$$W_{i}∼i.i.d\;\mathrm{Gamma}\left(\exp\left(\theta_{1}^{0}\right)=1,\exp\left(\theta_{2}^{0}\right)=2\right)\;\;\mathrm{for}\;i=1,\ldots ,n$$

where $\theta_{1}^{0}=\ln\left(1\right)=0$ and $\theta_{2}^{0}=\ln\left(2\right)$. We exploit the first two moments of the Gamma distribution, i.e., $E\left[W_{i}\right]=\exp\left(\theta_{1}^{0}+\theta_{2}^{0}\right)$ and $E\left[W_{i}^{2}\right]=\exp\left(\theta_{1}^{0}+2\theta_{2}^{0}\right)+\exp\left(2\theta_{1}^{0}+2\theta_{2}^{0}\right)$ to conduct the score tests. Consequently, the moment vector is defined as

$$g\left(W_{i},\left(\theta_{1},\theta_{2}\right)\right)=\left[\begin{array}{c} W_{i}-\exp\left[\theta_{1}+\theta_{2}\right]\\ W_{i}^{2}-\exp\left[\theta_{1}+2\theta_{2}\right]-\exp\left[2\theta_{1}+2\theta_{2}\right] \end{array}\right]$$

and it satisfies the moment restrictions in (1) for $\theta =\theta^{0}={\left(\theta_{1}^{0\prime },\theta_{2}^{0\prime }\right)}^{\prime }$. The Jacobian does not depend on $W_{i}$, so the implied probabilities are not involved in its estimation. The elements of the moment vector $g(W_{i};\theta^{0})$ are skewed. Indeed, the skewness of the first element is 2, while that of the second element is approximately 6.6. Moreover, the two elements of the moment vector are strongly leptokurtic with fourth moments equal to 144 (kurtosis = 9) and 8,982,528 (kurtosis = 87.7), respectively. Hence, the estimation of the variance might be problematic and, therefore, appropriate weighting for the estimator of the variance matrix might be crucial.

### 4.2. Results

There is no weak identification issue in this design. Hence, without the fear of over-rejection of the truth, according to the first-order asymptotics, one could plug in the restricted GMM estimator of $\theta_{2}$ in the second-step test statistic ${\mathrm{LM}}_{1.2,n}\left(.\right)$ instead of minimizing the test statistic ${\mathrm{LM}}_{1.2,n}\left(.\right)$ over values of $\theta_{2}$ in the first-step confidence interval. This is similar in spirit to the score test of Newey and West [6]. Taking advantage of the C(α) form of ${\mathrm{LM}}_{1.2,n}\left(.\right)$’s asymptotic invariance to $\sqrt{n}$-local deviation of $\theta_{2}$ from $\theta_{2}^{0}$, we plug in the computationally convenient restricted GMM estimator of $\theta_{2}$ in ${\mathrm{LM}}_{1.2,n}\left(.\right)$. We consider this plug-in version of the score test for three popular choices: (i) $\pi^{G}\left(.\right)=\pi^{V}\left(.\right)=1/n$; (ii) $\pi^{G}\left(.\right)=\pi^{V}\left(.\right)={\hat{\pi }}^{\left(1\right)}\left(.\right)$, i.e., the EEL implied probabilities; and (iii) $\pi^{G}\left(.\right)=\pi^{V}\left(.\right)={\hat{\pi }}^{\left(-1\right)}\left(.\right)$, i.e., the EL implied probabilities. We similarly consider each of these choices for the refined projection score test with two choices τ=1% and τ=5% for the first-step confidence interval. Asymptotic theory says that all tests considered here are asymptotically equivalent and efficient in this case.

To explore the finite-sample properties of the tests, we run 5000 Monte Carlo trials for the sample sizes n=100 and 1000. The theoretical size is α=5% for all tests. Table 1 contains the rejection rate of the null $H_{0}:\theta =\theta_{10}$ of all these tests for a grid of deviations from the null, i.e., $\theta_{10}-\theta_{1}^{0}$. The columns contain rejection rates for the plug-in score test and our refined test with two values of τ, τ=1%, 5%. The row with $\theta_{10}-\theta_{1}^{0}=0$ corresponds to the empirical size of the tests.

First, we analyze the size. We see that the plug-in version of the score test for all three choices of $\pi^{G}\left(.\right),\pi^{V}\left(.\right)$ over-rejects the true null. Over-rejection goes down for the choices $\pi^{G}\left(.\right)=\pi^{V}\left(.\right)=1/n$ and $\pi^{G}\left(.\right)=\pi^{V}\left(.\right)={\hat{\pi }}^{\left(-1\right)}\left(.\right)$ when the sample size increases to n=1000. However, the refined projection version of the score test for all three choices largely solves this problem of the over-rejection of the truth even when n=100. Importantly, we see that the choice of τ=1% versus τ=5% for the refined projection does not much affect the finite-sample rejection rate of the truth under this strong identification setup.

Moving to the discussion of power, we see that the refined projection test has good power in small samples. Now, comparing the choices $\pi^{G}\left(.\right)=\pi^{V}\left(.\right)=1/n$, $\pi^{G}\left(.\right)=\pi^{V}\left(.\right)={\hat{\pi }}^{\left(1\right)}\left(.\right)$, $\pi^{G}\left(.\right)=\pi^{V}\left(.\right)={\hat{\pi }}^{\left(-1\right)}\left(.\right)$, we see that the finite sample power of the third choice, i.e., EL, is much better than that of the other two. The lower power in small samples for the choice $\pi^{G}\left(.\right)=\pi^{V}\left(.\right)=1/n$ supports that orthogonalization by the implied probabilities in the variance matrix estimator is important for power. However, do note that the $\pi^{G}\left(.\right)=\pi^{V}\left(.\right)={\hat{\pi }}^{\left(1\right)}\left(.\right)$ (EEL) delivers the worst power in spite of the orthogonalization by the implied probabilities in the variance matrix estimator. This happens because the EEL implied probabilities can be negative, which rules out the positive (semi-) definiteness of the variance estimator and, in turn, leads to an unduly small ${\mathrm{LM}}_{1.2,n}\left(.\right)$ under false null hypotheses. The shrinkage of the EEL implied probabilities to make them positive, as suggested in Antoine et al. [47] and extensively used in Chaudhuri and Renault [9], can alleviate this problem of poor power to some extent but is not investigated here.

The refined projection test with the EL implied probabilities is the clear winner in terms of size and power. Its superiority is more prominent in the smaller sample, where it matters more.

Another Monte Carlo experiment using a linear instrumental variables regression confirms the good size and power of our test (the results are available from the authors upon request).

## 5. Application to the Impact of Veteran Status on Earnings

Following Chaudhuri and Rose [16], we propose to estimate the effect of the veteran status on future earnings for Vietnam war veterans in the United States by running an instrumental variables regression of log annual earnings on the dummy variable veteran status and a variety of control variables related to both earnings and veteran status. One important variable which influences earnings is years of schooling. However, since schooling is related to some unobservable variable (“ability”) that is related to both earnings and veteran status, it is obviously endogenous. So, we wish to estimate a regression of the log earnings on both veteran status and schooling. (The causal question in this empirical illustration is a difficult one due to the nature of the relationship between veteran status and schooling. First, the veteran status can help increase the years of schooling because of the subsidy provided by the GI Bill. Hence, schooling can be a mediator through which the veteran status affects wages. Second, the draft avoidance behavior of individuals was often enacted by enrolling in college and thereby increasing years of schooling. That is, the decisions to join the military and for the continuation of schooling were often made simultaneously. A more complete analysis is beyond the scope of this paper.) Given both regressors are endogenous, we need to use instrumental variables.

Angrist [17,48] used the Vietnam Era draft lottery that determined the draft eligibility of individuals, to instrument for an individual’s veteran status in the Vietnam war. A popular choice of instrument for schooling since Card [18,49,50] has been the presence of colleges in the neighborhood of where the individual grew up. Following these seminal references, we use four instrumental variables: (i) the lottery number assigned to the individual based on their date of birth, (ii) the lottery ceiling for the year when this individual attained draft age, (iii) a dummy variable indicating the presence of a 4 year accredited public college, and (iv) a dummy variable indicating the presence of a 4 year accredited private college in the neighborhood of the individual’s residence in 1966.

Partialling out the control variables from the system by taking the residuals from a regression of the concerned variables on those controls and the intercept, we focus on the instrumental variables regression model

$$y_{i}=X_{1i}\theta +X_{2i}\theta_{2}+u_{i}$$

with moment vector

$$g\left(W_{i};\theta \right)=Z_{i}\left(y_{i}-X_{1i}\theta_{1}-X_{2i}\theta_{2}\right)$$

where $y_{i},X_{1i},X_{2i}$ denote the residuals from the regression on the controls and the intercept of the variables log earnings, veteran status, and years of schooling, respectively, and $Z_{i}$ is the 4×1 vector of instruments such that $E\left(Z_{i}u_{i}\right)=0$.

We use the same data (the dataset is available on https://saraswata.research.mcgill.ca/MC_SC_Data.xlsx, accessed on 2 February 2025) as in Chaudhuri and Rose [16], which were obtained from the National Longitudinal Survey of Young Men. The sample includes 1080 (i.e., 39%) veterans and 1674 non-veterans. In this dataset, the instruments are weak for both veteran status and schooling with the first stage *F* statistic equal to 8.46 and 2.53, respectively.

Using these data, Chaudhuri and Rose [16] implemented a variety of plug-in methods, namely, the subset-K, subset-KJ and subset-CLR tests, and obtained a significant (at the 5% level) negative effect of the veteran status. However, these tests are not reliable in the presence of weak identification as shown by Guggenberger et al. [13] and Andrews [26].

The only genuinely weak-identification robust method used in Chaudhuri and Rose [16] is the so-called subset-Anderson–Rubin test proposed by Guggenberger et al. [13]. The subset-AR test lead to a 90% confidence interval for the coefficient of the veteran status whose upper bound is approximately 0.095, signifying that rather large positive effects of veteran status—100(exp(0.095)−1)=9.97% increase in wage—cannot be ruled out. The lower bound of the subset-AR confidence interval asymptotes to −∞, which is a consequence of weak identification. The inclusion of positive values in the confidence renders this test inconclusive.

The subset-AR test can be conservative when the effective number of over-identifying restrictions (the number of instruments minus the dimension of $\theta_{2}$, in this case 4−1=3) is larger than the number of restrictions in the null (in this case, 1) being tested. Therefore, a priori, there is reason to believe that the refined projection test, that is, the efficient test under strong identification but also robust to weak identification, might alter the conclusion of the subset-AR test.

Indeed, this is what we find with the refined projection test using EL implied probabilities $\pi^{G}\left(.\right)=\pi^{V}\left(.\right)={\hat{\pi }}^{\left(-1\right)}\left(.\right)$. This confidence interval also includes implausibly large negative values (consequence of weak identification); however, its upper bound is less than zero, supporting the hypothesis that the veteran effect is negative.

**Figure 1:**
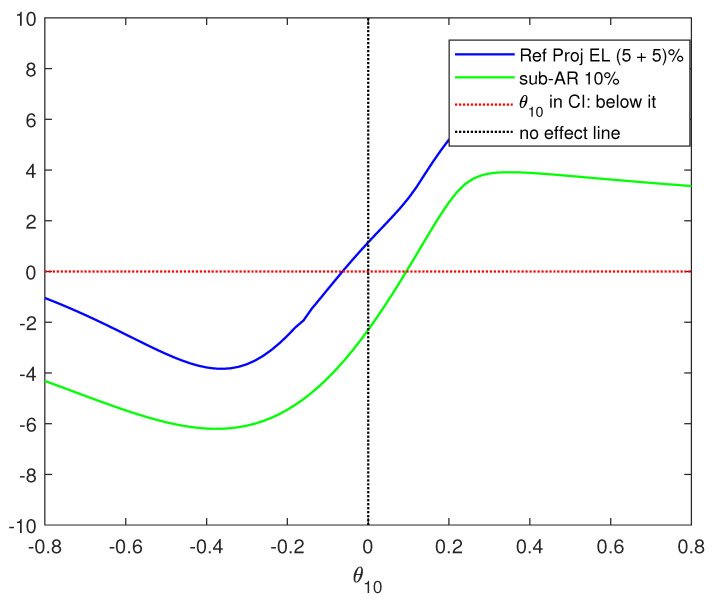
The values of $\theta_{10}$ below the horizontal line are included in the confidence interval obtained by inverting the refined projection test (blue line) and the subset-AR test (green line).

For a visual illustration, Figure 1 presents two plots against various values of $\theta_{10}$ of $H_{0}:\theta_{1}=\theta_{10}$—(i) the subset-AR statistic minus the $\chi_{3}^{2}\left(1-0.1\right)$, i.e., the tests statistic minus the 10% critical value for the subset-AR test, and (ii) the second step test statistic for the refined projection test minus the second step critical value, i.e., $\inf_{\theta_{2}\in C_{H_{0}}\left(\theta_{2},1-\tau \right)}{\mathrm{LM}}_{1.2,n}\left(\theta_{10},\theta_{2},\pi^{G}\left(\theta_{10},\theta_{2}\right),\pi^{V}\left(\theta_{10},\theta_{2}\right)\right)-\chi_{1}^{2}\left(1-\alpha \right)$ for the choice τ=α=0.05. We take the function plotted for (ii) as +∞ if the first-step confidence interval is empty (that automatically rejects $H_{0}:\theta_{1}=\theta_{10}$ without requiring the second step). The values $\theta_{10}$ for which these two plots are below the horizontal red dotted line at zero are those that are included in the confidence interval for the respective tests. The vertical black dotted line is the zero effect line. Inclusion of the blue or the green line in the south-east quadrant of the graph means the positive effect is not ruled out by the concerned test. We see that while the CI of the subset-AR test includes positive values, that of our refined test includes only negative values which permits to conclude that the veteran effect is negative.

## 6. Conclusions

In this paper, we propose a two-step approach for testing the subvectors of parameters in models characterized by a vector of moment restrictions. The first step is based on an identification robust confidence interval of the parameter, while the second relies on a score test. We show the advantages of using the implied probabilities obtained from the Information Theory criteria to estimate the Jacobian and variance matrix present in our score tests. These tests exploit efficiently the information content of the moment conditions. As a result, these tests have an empirical size close to the theoretical size and their power is good. The resulting confidence intervals are more reliable than those from alternative tests in the presence of skewness and/or weak identification. The theoretical properties of our tests are derived for all the elements of the Cressie–Read family, including the Kullback–Leibler Information Criterion. Finally, the empirical application brings evidence that veterans have lower earnings than comparable non-veterans.

## Figures and Tables

**Table 1:** Finite-sample rejection rate (in %) of score tests for $H_{10}:\theta_{1}=\theta_{10}$ with nominal level α=5%. The asymptotic size of the refined projection test cannot exceed α+τ.

Nominal Level		Plug-in and Refined Projection Score Tests with τ=5% and τ=1%								
α=5%		$\pi^{G}\left(.\right)=\pi^{V}\left(.\right)=1/n$			$\pi^{G}\left(.\right)=\pi^{V}\left(.\right)={\hat{\pi }}^{\left(1\right)}\left(.\right)$: EEL			$\pi^{G}\left(.\right)=\pi^{V}\left(.\right)={\hat{\pi }}^{\left(-1\right)}\left(.\right)$: EL		
n	$\theta_{10}-\theta_{1}^{0}$	Plug-in	τ=5%	τ=1%	Plug-in	τ=5%	τ=1%	Plug-in	τ=5%	τ=1%
100	−1	99.7	99.3	98.4	97.5	96.3	95.2	99.5	99.4	98.9
100	−0.8	98.6	96.9	93.7	92.5	89.5	87.0	97.6	96.7	95.2
100	−0.6	93.4	87.7	79.2	78.4	72.3	67.3	91.2	88.7	83.5
100	−0.4	76.2	64.0	50.4	50.9	42.9	37.6	72.6	64.9	55.4
100	−0.2	42.1	28.6	18.1	20.3	15.2	12.2	39.1	29.3	21.3
100	0	10.6	6.1	2.9	7.8	5.6	4.6	13.0	7.7	5.4
100	0.2	6.6	6.1	6.0	21.8	18.6	17.9	26.1	20.5	20.1
100	0.4	34.4	34.4	34.4	57.4	50.8	48.5	68.4	63.8	63.6
100	0.6	74.4	74.4	74.4	76.4	66.5	61.6	94.7	93.4	93.4
100	0.8	92.3	92.3	92.3	62.5	49.0	43.1	99.8	99.8	99.8
100	1	96.2	96.2	96.2	37.8	23.2	18.5	100.0	100.0	100.0
1000	−0.3162	99.3	99.3	99.1	99.3	97.6	92.9	97.1	96.1	94.9
1000	−0.253	96.9	96.3	95.7	97.6	93.4	84.7	91.6	88.1	86.1
1000	−0.1897	86.9	84.9	83.1	91.2	81.5	68.4	76.4	71.1	67.1
1000	−0.1265	60.5	57.3	53.8	73.1	56.9	41.4	48.1	42.3	37.5
1000	−0.0632	25.6	23.2	20.6	39.5	24.7	14.8	18.9	14.9	12.4
1000	0	6.2	5.6	4.8	11.9	6.1	3.1	6.8	4.8	4.0
1000	0.0632	11.4	11.4	11.3	15.8	7.4	5.8	21.3	18.8	18.8
1000	0.1265	45.1	45.1	45.1	33.2	16.0	13.0	61.1	58.2	58.2
1000	0.1897	85.6	85.6	85.6	24.8	13.3	11.1	92.0	90.8	90.8
1000	0.253	98.5	98.5	98.5	8.9	5.1	4.3	99.5	99.4	99.4
1000	0.3162	100.0	100.0	100.0	1.5	1.0	0.8	100.0	100.0	100.0

## Data Availability

Data are contained within the article.

## Appendix A

### Appendix A.1. Assumptions Involving Weak Identification

All the following assumptions are discussed in detail in Chaudhuri and Zivot [14]. Without loss of generality, we group the parameters into weakly and strongly identified parameters. For j=w,s, let $\nu_{j}=\nu_{1j}+\nu_{2j}$, $\theta_{j}={\left(\theta_{1j}^{\prime },\theta_{2j}^{\prime }\right)}^{\prime }$ and $\Theta_{j}=\Theta_{1j}\times \Theta_{2j}$. This notation denotes the weakly identified parameters as $\theta_{w}$ and the strongly identified parameters as $\theta_{s}$. The true values are, when convenient, regrouped as $\theta_{0w}={\left(\theta_{01w}^{\prime },\theta_{02w}^{\prime }\right)}^{\prime }$ and $\theta_{0s}={\left(\theta_{01s}^{\prime },\theta_{02s}^{\prime }\right)}^{\prime }$, respectively. When necessary, N⊂Θ and $N_{r}\subset \Theta_{r}$ are generically used to denote non-shrinking open neighborhoods of $\theta_{0}$ and $\theta_{0r}$ for r=1w,1s,2w,2s,w,s,1,2, respectively. Define $\tilde{N}:=N_{w}\times N_{1s}\times \Theta_{2s}$.

**Assumption** **Θ:** [*partition of parameter space*]

For l=1,2, let $\Theta_{l}=\Theta_{lw}\times \Theta_{ls}$ and for j=w,s, let $\theta_{lj}^{0}\in$ interior $\left(\Theta_{lj}\right)$, where $\Theta_{lj}\subset R^{\nu_{lj}}$ is compact.

**Assumption** **SW:** [*characterization of strong/weak identification*]

$E\left[{\bar{g}}_{n}\left(\theta \right)\right]={\tilde{m}}_{n}\left(\theta \right)/\sqrt{n}+m\left(\theta_{s}\right)$ where

(a) ${\tilde{m}}_{n}\left(\theta \right):\Theta \mapsto R^{d_{g}}$ is such that ${\tilde{m}}_{n}\left(\theta \right)\to \tilde{m}\left(\theta \right)$ uniformly for $\theta \in \tilde{N}$ where $\tilde{m}\left(\theta \right)$ is bounded and continuous and $\tilde{m}\left(\theta_{0}\right)=0$. For $\theta \in \tilde{N}$, ${\tilde{M}}_{n}\left(\theta \right):=\partial {\tilde{m}}_{n}\left(\theta \right)/\partial \theta^{\prime }$, ${\tilde{M}}_{n}\left(\theta \right)\to \tilde{M}\left(\theta \right)$ uniformly. $\tilde{M}\left(\theta \right)=\left[{\tilde{M}}_{1w}\left(\theta \right),{\tilde{M}}_{1s}\left(\theta \right),{\tilde{M}}_{2w}\left(\theta \right),{\tilde{M}}_{2s}\left(\theta \right)\right]$ where, for l=1,2 and j=w,s, the $k\times \nu_{lj}$ matrix ${\tilde{M}}_{lj}\left(\theta \right)$ is bounded and continuous.
(b) $m\left(\theta_{s}\right):\Theta_{s}\mapsto R^{d_{g}}$ is a continuous function and $m(\theta_{s})=0$ if and only if $\theta_{s}=\theta_{s}^{0}$. For $\theta_{s}\in N_{1s}\times \Theta_{2s}$, $M\left(\theta_{s}\right):=\partial m\left(\theta_{s}\right)/\partial \theta_{s}^{\prime }$ is bounded and continuous. $M(\theta_{0s})$ has full column rank. Here, $M\left(\theta_{s}\right)=\left[M_{1}\left(\theta_{s}\right),M_{2}\left(\theta_{s}\right)\right]$ where $M_{l}\left(\theta_{s}\right):=\partial m\left(\theta_{s}\right)/\partial \theta_{ls}^{\prime }$ for l=1,2.

**Assumption D:** [*assumptions on the moment vector and its derivative*]

D1. ${\bar{G}}_{n}\left(\theta \right):=\partial {\bar{g}}_{n}\left(\theta \right)/\partial \theta^{\prime }=\left[G_{1wn}\left(\theta \right),G_{1sn}\left(\theta \right),G_{2wn}\left(\theta \right),G_{2sn}\left(\theta \right)\right]=E\left[{\bar{G}}_{n}\left(\theta \right)\right]+o_{p}\left(1\right)$ uniformly for $\theta \in \tilde{N}$ where $E\left[{\bar{G}}_{n}\left(\theta \right)\right]=\partial E\left[{\bar{g}}_{n}\left(\theta \right)\right]/\partial \theta^{\prime }={\tilde{M}}_{n}\left(\theta \right)/\sqrt{n}+\left[0,M_{1}\left(\theta_{s}\right),0,M_{2}\left(\theta_{s}\right)\right]$ by imposing interchangeability of the order of differentiation and integration (and from Assumption SW).
D2. $\sqrt{n}\left[{\bar{g}}_{n}^{\prime }\left(\theta_{0}\right),vec^{\prime }\left({\bar{G}}_{wn}\left(\theta_{0}\right)-E\left[{\bar{G}}_{wn}\left(\theta^{0}\right)\right]\right)\right]\overset{d}{\to }\left[\Psi_{g}^{T},\Psi_{w}^{T}\right]$ where (the partition of $\Psi_{w}\left(\theta \right)={\left[\Psi_{1w}^{\prime }\left(\theta \right),\Psi_{2w}^{\prime }\left(\theta \right)\right]}^{\prime }$, $\Sigma_{gw}\left(\theta \right)=\left[\Sigma_{g1}\left(\theta \right),\Sigma_{g2}\left(\theta \right)\right]=\Sigma_{wg}^{\prime }\left(\theta \right)$ and $\Sigma_{ww}\left(\theta \right)={\left(\Sigma_{ll^{\prime }}\left(\theta \right)\right)}_{l,l^{\prime }=1,2}$ is conformable to the partition of $\theta_{w}={\left(\theta_{1w}^{\prime },\theta_{2w}^{\prime }\right)}^{\prime }$, the partition of the weakly identified elements of θ into those from $\theta_{1}$ and $\theta_{2}$, respectively)
  $$\left[\begin{array}{c} \Psi_{g}\\ \Psi_{w} \end{array}\right]∼N\left(0,\Sigma \left(\theta_{0}\right)=\left[\begin{array}{cc} \underset{k\times k}{\Sigma_{gg}\left(\theta^{0}\right)}\equiv V\left(\theta^{0}\right) & \underset{k\times k\nu_{w}}{\Sigma_{gw}\left(\theta^{0}\right)}\\ \underset{k\nu_{w}\times k}{\Sigma_{wg}\left(\theta^{0}\right)} & \underset{k\nu_{w}\times k\nu_{w}}{\Sigma_{ww}\left(\theta^{0}\right)} \end{array}\right]\right).$$
  $\Sigma_{gg}\left(\theta \right)$ is bounded, continuous, and positive definite. Refining Assumption 2 and with the obvious correspondence of notation between the *V*’s and the Σ’s that specify what the estimators are, we also make the following assumptions: ${\hat{\Sigma }}_{gg}\left(\theta \right)\overset{p}{\to }\Sigma_{gg}\left(\theta \right)$ uniformly for $\theta \in \tilde{N}$. $\Sigma_{wg}\left(\theta \right)$ is bounded and continuous. ${\hat{\Sigma }}_{wg}\left(\theta \right):={\left[{\hat{\Sigma }}_{1g}^{\prime }\left(\theta \right),\ldots ,{\hat{\Sigma }}_{\nu_{1w},g}^{\prime }\left(\theta \right),{\hat{\Sigma }}_{\nu_{1}+1,g}^{\prime }\left(\theta \right),\ldots ,{\hat{\Sigma }}_{\nu_{1}+\nu_{2w},g}^{\prime }\left(\theta \right)\right]}^{\prime }$$\overset{p}{\to }\Sigma_{wg}\left(\theta \right)$ uniformly for θ∈N. (It is worth noting that the $d_{g}\times d_{g}$ matrix ${\hat{\Sigma }}_{lg}\left(\theta^{0}\right)\overset{p}{\to }\Sigma_{lg}\left(\theta^{0}\right)=\mathrm{Asym}.\mathrm{Cov}\left(n^{-1/2}\partial {\bar{g}}_{n}\left(\theta^{0}\right)/\partial \theta_{l},n^{-1/2}{\bar{g}}_{n}\left(\theta^{0}\right)\right)$ where $\theta_{l}$ is the *l*-th element ($l=1,\ldots ,\nu_{1w},\nu_{1}+1,\ldots ,\nu_{1}+\nu_{2w}$) of θ.) For $l=\nu_{1w}+1,\ldots ,\nu_{1},\nu_{1}+\nu_{2w}+1,\ldots ,\nu$ the $d_{g}\times d_{g}$ matrices ${\hat{\Sigma }}_{lg}\left(\theta \right)$ are such that ${\hat{\Sigma }}_{lg}\left(\theta \right){\hat{\Sigma }}_{gg}^{-1}\left(\theta \right)=O_{p}\left(1\right)$ uniformly for θ∈N.

### Appendix A.2. Proofs

In the proofs, we use the notation $g_{i}\left(\theta \right)=g\left(W_{i};\theta \right).$

**Proof of Proposition** **1:**
* *
(A) A mean-value expansion of the RHS of the (approximate) first-order condition of the maximization problem in (5) gives,

(A1) $$\begin{array}{ccc} o_{P}\left(\frac{1}{\sqrt{n}}\right) & = & \frac{1}{n}\sum_{i=1}^{n}\rho_{1}\left(\lambda_{\rho ,n}^{\prime }\left(\theta \right)g_{i}\left(\theta \right)\right)g_{i}\left(\theta \right)\\ & = & \frac{1}{n}\sum_{i=1}^{n}\rho_{1}\left(0\right)g_{i}\left(\theta \right)+\frac{1}{n}\sum_{i=1}^{n}\rho_{2}\left(0\right)g_{i}\left(\theta \right)g_{i}^{\prime }\left(\theta \right)\lambda_{\rho ,n}\left(\theta \right)+R_{\lambda ,n}\left(\theta \right)\\ & = & -{\bar{g}}_{n}\left(\theta \right)-{\hat{\Omega }}_{n}\left(\theta \right)\lambda_{\rho ,n}\left(\theta \right)+R_{\lambda ,n}\left(\theta \right), \end{array}$$

where ${\bar{v}}_{i}$ are the mean values satisfying $|{\bar{v}}_{i}\left|\leq \right|\lambda_{\rho ,n}^{\prime }\left(\theta \right)g_{i}\left(\theta \right)|$ for all i=1,…,n, and the remainder term $R_{\lambda ,n}\left(\theta \right)=\frac{1}{n}\sum_{i=1}^{n}\left[\rho_{2}\left({\bar{v}}_{i}\right)-\rho_{2}\left(0\right)\right]g_{i}\left(\theta \right)g^{\prime }\left(W_{i};\theta \right)\lambda_{\rho ,n}\left(\theta \right)$. If we could ignore the contribution of $R_{\lambda ,n}\left(\theta \right)$ in (A1), we would obtain

$$\lambda_{\rho ,n}\left(\theta \right)=-{\hat{\Omega }}_{n}^{-1}\left(\theta \right){\bar{g}}_{n}\left(\theta \right)+{\hat{\Omega }}_{n}^{-1}\left(\theta \right)\times o_{P}\left(\frac{1}{\sqrt{n}}\right)=O_{P}\left(\frac{1}{\sqrt{n}}\right).$$

${\hat{\Omega }}_{n}\left(\theta \right)$ and ${\hat{\Omega }}_{n}^{-1}\left(\theta \right)$ are assumed to be $O_{P}\left(1\right)$ by assumption A2 and ${\bar{g}}_{n}\left(\theta \right)=O_{P}\left(n^{-1/2}\right)$ by assumption A1 (i) and (iv). For this reason, if we can show that $∥R_{\lambda ,n}\left(\theta \right)∥=o_{P}\left(n^{-1/2}\right),$ then it will be sufficient to establish result (A). This is what we prove next:

(A2) $$\begin{array}{ccc} ∥R_{\lambda ,n}\left(\theta \right)∥ & = & ∥\left(\frac{1}{n}\sum_{i=1}^{n}\left[\rho_{2}\left({\bar{v}}_{i}\right)-\rho_{2}\left(0\right)\right]g_{i}\left(\theta \right)g_{i}^{\prime }\left(\theta \right)\right)\lambda_{\rho ,n}\left(\theta \right)∥\\ & \leq & ∥\frac{1}{n}\sum_{i=1}^{n}\left[\rho_{2}\left({\bar{v}}_{i}\right)-\rho_{2}\left(0\right)\right]g_{i}\left(\theta \right)g_{i}^{\prime }\left(\theta \right)∥\times ∥\lambda_{\rho ,n}\left(\theta \right)∥\\ & \leq & \max_{1\leq i\leq n}|\rho_{2}\left({\bar{v}}_{i}\right)-\rho_{2}\left(0\right)|\times ∥\frac{1}{n}\sum_{i=1}^{n}g_{i}\left(\theta \right)g_{i}^{\prime }\left(\theta \right)∥\times ∥\lambda_{\rho ,n}\left(\theta \right)∥\\ & \leq & b\times \max_{1\leq i\leq n}|{\bar{v}}_{i}|\times \left(∥V\left(\theta \right)∥+o_{p}\left(1\right)\right)\times ∥\lambda_{\rho ,n}\left(\theta \right)∥\\ & \leq & b\times \max_{1\leq i\leq n}|g_{i}^{\prime }\left(\theta \right)\lambda_{\rho ,n}\left(\theta \right)|\times b_{\max}\left(\theta \right)\times ∥\lambda_{\rho ,n}\left(\theta \right)∥\\ & \leq & b\times \max_{1\leq i\leq n}∥g_{i}\left(\theta \right)∥\times b_{max}\left(\theta \right)\times {∥\lambda_{\rho ,n}\left(\theta \right)∥}^{2}\\ & \leq & b\times b_{\max}\left(\theta \right)\times o_{p}\left(\sqrt{n}\right)\times {∥\lambda_{\rho ,n}\left(\theta \right)∥}^{2}=o_{P}\left(n^{-1/2}\right), \end{array}$$

by the repeated use of Cauchy–Schwarz and triangle inequalities and because $\max_{1\leq i\leq n}∥g_{i}\left(\theta \right)∥=o_{P}\left(\sqrt{n}\right)$ and $∥\lambda_{\rho ,n}\left(\theta \right)∥=O_{P}\left(n^{-1/2}\right)$. Therefore, result (A) follows. (B) Expanding the numerator and denominator of the RHS of (6) around 0, and using the result obtained in (A), we obtain for any given i=1,…,n

(A3) $$\begin{array}{ccc} \pi_{\rho ,i,n}\left(\theta \right) & = & \frac{\frac{1}{n}\left[\rho_{1}\left(0\right)+\rho_{2}\left(0\right)\lambda_{\rho ,n}^{\prime }\left(\theta \right)g_{i}\left(\theta \right)+\left\{\rho_{2}\left({\bar{v}}_{i}\right)-\rho_{2}\left(0\right)\right\}\lambda_{\rho ,n}^{\prime }g_{i}\left(\theta \right)\right]}{\frac{1}{n}\sum_{j=1}^{n}\left[\rho_{1}\left(0\right)+\rho_{2}\left(0\right)\lambda_{\rho ,n}^{\prime }g_{i}\left(\theta \right)+\left\{\rho_{2}\left({\bar{v}}_{j}\right)-\rho_{2}\left(0\right)\right\}\lambda_{\rho ,n}^{\prime }g\left(W_{j};\theta \right)\right]}\\ & = & \frac{\frac{1}{n}\left[\rho_{1}\left(0\right)-\rho_{2}\left(0\right)g_{i}^{\prime }\left(\theta \right)\left\{{\hat{\Omega }}_{n}^{-1}\left(\theta \right){\bar{g}}_{n}\left(\theta \right)+o_{P}\left(n^{-1/2}\right)\right\}+\left\{\rho_{2}\left({\bar{v}}_{i}\right)-\rho_{2}\left(0\right)\right\}\lambda_{\rho ,n}^{\prime }\left(\theta \right)g_{i}\left(\theta \right)\right]}{\frac{1}{n}\sum_{j=1}^{n}\left[\rho_{1}\left(0\right)-\rho_{2}\left(0\right)g_{i}^{\prime }\left(\theta \right)\left\{{\hat{\Omega }}_{n}^{-1}\left(\theta \right){\bar{g}}_{n}\left(\theta \right)+o_{P}\left(n^{-1/2}\right)\right\}+\left\{\rho_{2}\left({\bar{v}}_{j}\right)-\rho_{2}\left(0\right)\right\}\lambda_{\rho ,n}^{\prime }\left(\theta \right)g\left(W_{j};\theta \right)\right]}\\ & = & \frac{\frac{1}{n}\left[1-{\left(g_{i}\left(\theta \right)-{\bar{g}}_{n}\left(\theta \right)\right)}^{\prime }{\hat{\Omega }}_{n}^{-1}\left(\theta \right){\bar{g}}_{n}\left(\theta \right)\right]+R_{NUM,i,n}}{1-{\bar{g}}_{n}^{\prime }\left(\theta \right){\hat{\Omega }}_{n}^{-1}\left(\theta \right){\bar{g}}_{n}\left(\theta \right)+R_{DEN,n}} \end{array}$$

where the remainder terms in the numerator and the denominator are given by

$$\begin{array}{ccc} R_{NUM,i,n}:= & & \frac{1}{n}\left\{\rho_{2}\left({\bar{v}}_{i}\right)-\rho_{2}\left(0\right)\right\}\lambda_{\rho ,n}^{\prime }\left(\theta \right)g_{i}\left(\theta \right)-\frac{1}{n}\rho_{2}\left(0\right)g^{\prime }\left(W_{i};\theta \right)\times o_{P}\left(n^{-1/2}\right)+\frac{1}{n}{\bar{g}}_{n}^{\prime }\left(\theta \right){\hat{\Omega }}_{n}^{-1}\left(\theta \right){\bar{g}}_{n}\left(\theta \right),\\ R_{DEN,n}:= & & \frac{1}{n}\sum_{j=1}^{n}\left[\left\{\rho_{2}\left({\bar{v}}_{j}\right)-\rho_{2}\left(0\right)\right\}\lambda_{\rho ,n}^{\prime }\left(\theta \right)g\left(W_{j};\theta \right)-\rho_{2}\left(0\right)g^{\prime }\left(W_{i};\theta \right)\times o_{P}\left(n^{-1/2}\right)\right]. \end{array}$$

It is important to note that *i* is given (fixed) in the remainder term $R_{NUM,i,n}$. Now following the same steps as in (A) to deal with the remainder term, we obtain for a given i=1,…,n

(A4) $$\begin{array}{ccc} |R_{NUM,i,n}| & \leq & \frac{1}{n}|\rho_{2}\left({\bar{v}}_{i}\right)-\rho_{2}\left(0\right)|\times ∥\lambda_{\rho ,n}\left(\theta \right)∥\times ∥g_{i}\left(\theta \right)∥+\frac{1}{n}∥g_{i}\left(\theta \right)∥\times o_{P}\left(\frac{1}{\sqrt{n}}\right)+\frac{1}{n}{\bar{g}}_{n}^{\prime }\left(\theta \right){\hat{\Omega }}_{n}^{-1}\left(\theta \right){\bar{g}}_{n}\left(\theta \right)\\ & \leq & \frac{1}{n}b\times |\lambda_{\rho ,n}^{\prime }\left(\theta \right)g_{i}\left(\theta \right)|\times ∥\lambda_{\rho ,n}\left(\theta \right)∥\times ∥g_{i}\left(\theta \right)∥+∥g_{i}\left(\theta \right)∥\times o_{P}\left(\frac{1}{n^{3/2}}\right)+\frac{1}{n}{∥{\bar{g}}_{n}\left(\theta \right)∥}^{2}\times b_{min}^{-1}\left(\theta \right)\\ & \leq & \frac{1}{n}b\times ∥\lambda_{\rho ,n}{\left(\theta \right)∥}^{2}\times ∥g_{i}{\left(\theta \right)∥}^{2}+∥g_{i}\left(\theta \right)∥\times o_{P}\left(\frac{1}{n^{3/2}}\right)+\frac{1}{n}{∥{\bar{g}}_{n}\left(\theta \right)∥}^{2}\times b_{min}^{-1}\left(\theta \right),\\ & = & O_{P}\left(\frac{1}{n^{1+1}}\right)\times O_{P}\left(1\right)+O_{P}\left(1\right)\times o_{P}\left(\frac{1}{n^{3/2}}\right)+O_{P}\left(\frac{1}{n^{1+1}}\right)\\ & = & o_{P}\left(\frac{1}{n^{3/2}}\right) \end{array}$$

because $∥g_{i}\left(\theta \right)∥=O_{P}\left(1\right)$ by A1(iii), $∥{\bar{g}}_{n}\left(\theta \right)∥=O_{P}\left(n^{-1/2}\right)$ by A1 (i) and (iv), and $\lambda_{\rho ,n}\left(\theta \right)=O_{P}\left(n^{-1/2}\right)$ by (A). Finally, we want to derive the order of magnitude of $|R_{DEN,n}|$. Using a similar technique as before, we obtain that

$$\begin{array}{ccc} |R_{DEN,n}| & \leq & \frac{1}{n}\left|\sum_{j=1}^{n}\left\{\rho_{2}\left({\bar{v}}_{j}\right)-\rho_{2}\left(0\right)\right\}\lambda_{\rho ,n}^{\prime }\left(\theta \right)g\left(W_{j};\theta \right)\right|+∥{\bar{g}}_{n}\left(\theta \right)∥\times o_{P}\left(n^{-1/2}\right)\\ & \leq & \max_{1\leq j\leq n}|\rho_{2}\left({\bar{v}}_{j}\right)-\rho_{2}\left(0\right)|\times ∥{\bar{g}}_{n}\left(\theta \right)∥\times ∥\lambda_{\rho ,n}\left(\theta \right)∥+∥{\bar{g}}_{n}\left(\theta \right)∥\times o_{P}\left(n^{-1/2}\right)\\ & \leq & b\times \max_{1\leq j\leq n}|\lambda_{\rho ,n}^{\prime }\left(\theta \right)g_{j}\left(\theta \right)|\times ∥{\bar{g}}_{n}\left(\theta \right)∥\times ∥\lambda_{\rho ,n}\left(\theta \right)∥+∥{\bar{g}}_{n}\left(\theta \right)∥\times o_{P}\left(n^{-1/2}\right)\\ & \leq & b\times \max_{1\leq j\leq n}∥g_{j}\left(\theta \right)∥\times ∥{\bar{g}}_{n}\left(\theta \right)∥\times ∥\lambda_{\rho ,n}{\left(\theta \right)∥}^{2}+∥{\bar{g}}_{n}\left(\theta \right)∥\times o_{P}\left(n^{-1/2}\right)\\ & = & o_{P}\left(n^{1/2-3/2}\right)+o_{P}\left(n^{-1}\right)=o_{P}\left(n^{-1}\right) \end{array}$$

because $\max_{1\leq j\leq n}∥g_{j}\left(\theta \right)∥=o_{P}\left(\sqrt{n}\right)$ by Assumption 1(ii), while by (A) we have $\lambda_{\rho ,n}\left(\theta \right)=O_{P}\left(n^{-1/2}\right)$. Moreover, ${\bar{g}}_{n}^{\prime }\left(\theta \right){\hat{\Omega }}_{n}^{-1}\left(\theta \right){\bar{g}}_{n}\left(\theta \right)$ in the denominator of (A3) is $O_{P}\left(n^{-1}\right)$ because $∥{\bar{g}}_{n}\left(\theta \right)∥=O_{P}\left(n^{-1/2}\right)$ as before. Therefore, the whole denominator of (A3) is $1+O_{P}\left(n^{-1}\right)$. Consequently, result (B) follows from (A3) and (A4).

**Proof of Proposition** **2:**
* *
(A) This result follows directly from the definition of the $\pi_{EEL,i,n}\left(\theta \right)$ and (vi). (B) Since our result in Proposition 1(B) is not uniform in i, we cannot appeal to $\max_{1\leq i\leq n}\left|\pi_{\rho ,i,n}\left(\theta \right)-\pi_{EEL,i,n}\left(\theta \right)\right|$ after applying the Cauchy–Schwarz inequality. Alternatively, we directly work with the expression of the difference $\left\{\pi_{\rho ,i,n}\left(\theta \right)-\pi_{EEL,i,n}\left(\theta \right)\right\}=R_{NUM,i}/(1+o_{P}\left(1\right)$ obtained in (A3). To simplify notations, we denote ${\tilde{Y}}_{i,n}:=Y_{i,n}-E\left[{\bar{Y}}_{n}\right]$, $g_{i}:=g_{i}\left(\theta \right)$, ${\bar{g}}_{n}:={\bar{g}}_{n}\left(\theta \right)$, ${\hat{\Omega }}_{n}:={\hat{\Omega }}_{n}\left(\theta \right)$ and $\lambda :=\lambda_{\rho ,n}\left(\theta \right)$. Accordingly, using Proposition 1(A), and assumptions A1 and A2, we obtain

$$\begin{array}{ccc} \; & & ∥\sqrt{n}\sum_{i=1}^{n}{\tilde{Y}}_{i,n}\left\{\pi_{\rho ,i,n}\left(\theta \right)-\pi_{EEL,i,n}\left(\theta \right)\right\}∥\\ \; & \leq & ∥\frac{1}{n}\sum_{i=1}^{n}\left\{\rho_{2}\left({\bar{v}}_{i}\right)-\rho_{2}\left(0\right)\right\}{\tilde{Y}}_{i,n}g_{i}^{\prime }\sqrt{n}\lambda ∥+o_{P}\left(1\right)\times ∥\frac{1}{n}\sum_{i=1}^{n}{\tilde{Y}}_{i,n}g_{i}^{\prime }∥+{\bar{g}}_{n}^{\prime }{\hat{\Omega }}_{n}^{-1}{\bar{g}}_{n}\times ∥\frac{1}{\sqrt{n}}\sum_{i=1}^{n}{\tilde{Y}}_{i,n}∥\\ \; & \leq & \sqrt{\frac{1}{n}\sum_{i=1}^{n}{\left\{\rho_{2}\left({\bar{v}}_{i}\right)-\rho_{2}\left(0\right)\right\}}^{2}}\times ∥\frac{1}{n}\sum_{i=1}^{n}{\tilde{Y}}_{i,n}g_{i}^{\prime }∥\times ∥\sqrt{n}\lambda ∥+o_{P}\left(1\right)\times ∥\frac{1}{n}\sum_{i=1}^{n}{\tilde{Y}}_{i,n}g_{i}^{\prime }∥+{\bar{g}}_{n}^{\prime }{\hat{\Omega }}_{n}^{-1}{\bar{g}}_{n}\times ∥\frac{1}{\sqrt{n}}\sum_{i=1}^{n}{\tilde{Y}}_{i,n}∥\\ \; & \leq & b\times \sqrt{\frac{1}{n}\sum_{i=1}^{n}{\left|\lambda^{\prime }g_{i}\right|}^{2}}\times ∥\frac{1}{n}\sum_{i=1}^{n}{\tilde{Y}}_{i,n}g_{i}^{\prime }∥\times ∥\sqrt{n}\lambda ∥+o_{P}\left(1\right)\times ∥\frac{1}{n}\sum_{i=1}^{n}{\tilde{Y}}_{i,n}g_{i}^{\prime }∥+{\bar{g}}_{n}^{\prime }{\hat{\Omega }}_{n}^{-1}{\bar{g}}_{n}\times ∥\frac{1}{\sqrt{n}}\sum_{i=1}^{n}{\tilde{Y}}_{i,n}∥\\ \; & \leq & b\times \sqrt{\frac{1}{n}\sum_{i=1}^{n}{∥g_{i}∥}^{2}}\times ∥\frac{1}{n}\sum_{i=1}^{n}{\tilde{Y}}_{i,n}g_{i}^{\prime }∥\times \sqrt{n}{∥\lambda ∥}^{2}+o_{P}\left(1\right)\times ∥\frac{1}{n}\sum_{i=1}^{n}{\tilde{Y}}_{i,n}g_{i}^{\prime }∥+{\bar{g}}_{n}^{\prime }{\hat{\Omega }}_{n}^{-1}{\bar{g}}_{n}\times ∥\frac{1}{\sqrt{n}}\sum_{i=1}^{n}{\tilde{Y}}_{i,n}∥\\ \; & = & O_{P}\left(1\right)\times O_{P}\left(1\right)\times O_{P}\left(n^{-1/2}\right)+o_{P}\left(1\right)\times O_{P}\left(1\right)+O_{P}\left(n^{-1}\right)\times O_{P}\left(1\right)=o_{P}\left(1\right), \end{array}$$

from the standard arguments, for example, $∥n^{-1}\sum_{i=1}^{n}{\tilde{Y}}_{i,n}g_{i}^{\prime }∥\leq ∥n^{-1}\sum_{i=1}^{n}{\tilde{Y}}_{i,n}{\left(g_{i}-{\bar{g}}_{n}\right)}^{\prime }∥+∥\left({\bar{Y}}_{n}-E\left[{\bar{Y}}_{n}\right]\right){\bar{g}}_{n}∥\leq ∥V_{Yg}∥+o_{P}\left(1\right)+O_{P}(n^{-1/2}∥V_{YY}∥)\times O_{P}(∥{\bar{g}}_{n}∥)=O_{P}\left(1\right)$.
